# Recovery of Health and Wellbeing in Rural Cancer Survivors Following Primary Treatment: Analysis of UK Qualitative Interview Data

**DOI:** 10.3390/nursrep12030046

**Published:** 2022-07-05

**Authors:** Florence Graham, Ros Kane, Mark Gussy, David Nelson

**Affiliations:** 1 Lincoln Medical School, College of Science, Universities of Nottingham and Lincoln, Lincoln LN6 7TS, UK; mzyfg2@nottingham.ac.uk; 2 School of Health and Social Care, University of Lincoln, Lincoln LN6 7TS, UK; rkane@lincoln.ac.uk; 3 Lincoln International Institute for Rural Health (LIIRH), University of Lincoln, Lincoln LN6 7TS, UK; mgussy@lincoln.ac.uk; 4 Macmillan Cancer Support, London SE1 7UQ, UK

**Keywords:** cancer survivorship, living with cancer, rural health, qualitative research, interview study, United Kingdom

## Abstract

**Purpose:** Rural cancer survivors have poorer experiences and health outcomes compared to their urban counterparts. There is limited research on the post-treatment experiences of UK cancer survivors residing in rural areas. This study aimed to provide an understanding of the specific challenges and opportunities faced by rural cancer survivors and to provide insight into how rurality influences experiences post-primary treatment, ultimately to inform service provision. **Methods:** A secondary analysis of in-depth interview transcripts (*n* = 16) from a wider study on self-management in cancer survivors was conducted. An adapted version of Foster and Fenlon’s recovery of health and wellbeing in cancer survivorship framework informed the data coding. **Results:** Health and wellbeing were interrupted by a variety of problem incidents, and the subsequent steps to recovery were influenced by pre-existing, personal, environmental, and healthcare factors. A prominent theme was support, both from local communities and family as well as from healthcare professionals, with many survivors feeling that their rural setting had a positive influence on their health and wellbeing. Close relationships with local GPs were seen as fundamental to supporting recovery. Access to healthcare was frequently mentioned as a challenge with an emphasis on lengthy travel times and limited bespoke support in rural areas. **Conclusions:** This study is novel in that it applied a well-established theoretical framework to a rich qualitative dataset on the lived experiences of rural cancer survivors. Rural residency influenced recovery from cancer both positively and negatively. **Implications for Cancer Survivors:** Future practitioners and policy makers should consider working with local communities to tailor interventions to the specific characteristics of the rural environment.

## 1. Introduction

In the UK in 2017, there were almost 370,000 new cancers diagnosed [1], and in 2010–2011, the cancer survival rates in England and Wales were 70% at one year, 54% at five years, and 50% at ten years post-diagnosis [2]. With cancer survival rates continuing to rise, tailoring care in line with cancer survivors’ needs is becoming increasingly important. 

Survivorship is the state of living with and beyond cancer, and it encompasses all the experiences people have as a result of a cancer diagnosis [3]. Macmillan Cancer Support, a national charity in Britain, defines survivors as ‘those not undergoing active treatment, and not within the terminal stages of cancer’ [4]. For this study, a cancer survivor was therefore defined as someone who has completed primary treatment within the last five years, who is now self-managing the medium- to longer-term physical, emotional, and social side effects of cancer, and who is not in receipt of palliative or end-of-life care. 

Cancer survivors are known to have a wide range of health needs following their treatment, ref. [5] and general practitioners (GPs) report a willingness but also a reticence to provide care to survivors due to little knowledge and confidence regarding their specific needs [6,7]. 

Recovery from cancer can present many issues for survivors, including but not limited to ongoing fatigue and pain, poor general health and wellbeing, , mental ill health, poor access to psychological support, digestive problems, an increased risk of other long-term conditions, isolation, sexual function problems, self-managing the acute side effects of treatment to a continuing fear of recurrence, a reduced quality of life, and needs related to returning to work [3,4,8,9,10,11,12]. The barriers to recovery include a loss of independence due to physical symptoms preventing travel and recreation; perceptions that once cancer treatment is completed, a survivor is back to normal; difficulty accessing self-management support; and psychosocial factors, such as a low self-confidence and feeling vulnerable. Facilitators include personal factors, such as proactiveness; forward planning; connecting with others via shared experiences; having an optimistic view in life; support from family, friends, employers, and co-workers; and using the Internet to support self-management [13]. 

Rural areas are defined as settlements with populations smaller than 10,000 people, and 9.7 million people in England live rurally [14]. This research examined the intersection between rurality and cancer survivorship [15,16,17]. A focus on rurality is important as internationally, rural populations have a lower rate of survival from cancer and report different experiences with healthcare systems and cancer recovery compared with their urban counterparts [9,18,19,20].

There is little research in the UK on rural populations experiencing cancer, and as survival rates are increasing and more people are living with and beyond cancer for several years [21], data on survivorship experience has become of paramount importance for policymakers and healthcare planners. Qualitative research is useful in gaining a detailed understanding of the range of situations and emotions a particular population faces, giving meaningful scoped data [22,23], and is especially applicable to complex specialist fields of research [24,25]. 

Given this dearth of literature from the UK, this study was designed to provide insight into how rurality influences cancer recovery and survivorship and sought to gain a better understanding of the specific challenges and opportunities faced by rural cancer survivors following primary treatment. Through the key themes identified in interview data, the study informs service improvement and guides future research around rurality and cancer recovery. 

### 1.1. Research Questions

- What are the specific challenges and opportunities faced by rural cancer survivors post-treatment?
- How does residing in a rural area influence recovery from cancer?

### 1.2. Rurality and Cancer Survivorship

There is a consensus within the extant literature that the provision of healthcare services tends to be poorer in rural areas. The logistical difficulties of providing specialist care to smaller, sparse populations and necessitating long-distance travel to larger towns or cities to receive adequate care; increased costs and a lack of resources; a need for higher staff-to-patient ratios and multidisciplinary input; difficulty accessing information; and poor communication and care coordination have all been identified as salient issues for service delivery [18,19,26,27,28,29,30,31]. Consequently, the accessibility to services tends to be lower in rural areas [15,18,19,27,28], and many people rely on informal social support from family and friends rather than formal support [19]. 

Those living in rural areas have a higher prevalence of comorbidities, more persistent symptoms and side effects of cancer and treatment [20,29,32], and overall higher excess risk for poorer health outcomes [15,16,26]. These outcomes are commonly attributed to lower socioeconomic statuses (SESs) and a lower health literacy in rural communities [32]. However, even taking these factors into account, rural populations have still been found to be 5% less likely to survive cancer, suggesting that it is rurality itself that may confer poorer outcomes [15]. Rural people with cancer also experience poorer mental health [18,32] as well as diminished physical capabilities, more physical inactivity, and lower functional wellbeing compared with their urban counterparts [18,20,26]. Additionally, they are less satisfied with their overall quality of life (QOL) [20] and more likely to report emotional distress [26]. 

There are, however, also positive elements of rural living; people who reside in rural areas report lower levels of severe distress, more independence, more positive thinking, and greater community engagement, as well as a higher self-efficacy for self-managing the consequences of cancer [28,33,34,35]. 

There is a lack of research into cancer recovery and survivorship in rural areas of the UK in particular, yet almost a fifth of the total UK population resides in rural areas. This study helps to develop a better understanding of the qualitative lived experiences of cancer survivors who reside in rural parts of the UK. The findings have the potential to inform future service deliveries to ensure that they are tailored to the needs of rural cancer survivors. 

## 2. Methods

The Consolidated Criteria for Reporting Qualitative Research (COREQ) guidelines were adhered to when reporting this research and the study results [36]. 

### 2.1. Study Design

This paper reports on the secondary analysis of a primary dataset collected as a component of a wider study [31,35,37]. The original study was conducted to explore and compare cancer survivors’ experiences of self-management following treatments in rural and urban areas [37] and aimed to understand the problems and opportunities presented by cancer survivorship from the points of view of those who experienced it firsthand. A qualitative interview-based approach was chosen as unstructured and semi-structured interviews can be useful to both researchers and participants in that interviewees find opportunities for validation and personal growth as well as self-reflection [38,39]. With the limited UK-specific qualitative research on rural cancer survivors [31], there is a pressing need to better understand their lived experiences as well as the individual contextual factors that can impact their experiences when recovering from cancer. 

Eligibility criteria for recruitment to the primary study are reported on in Table 1 below.

Transcripts from the wider study were considered eligible for this more in-depth secondary analysis if the participant lived in a rural area as designated by the UK Office for National Statistics (ONS) RUC2011 Rural Urban Classification [40]. Participants had previously been asked to provide their postcodes, and the ONS postcode directory look-up tool (https://onsdigital.github.io/postcode-lookup/ last accessed on 1 July 2022) was used to allocate each person to their RUC2011 category.

#### 2.1.1. Interviewing

In the primary study, thirty-four interviews were conducted face to face both at the host university and in participants’ homes as well as via telephone at the participants’ convenience. All were audio-recorded and transcribed verbatim and ranged in length from approximately 30 to 100 min. Participants were provided with an information sheet and consent form prior to the interview taking place, with an opportunity to ask any questions of the interviewer. Interviews were guided by a topic guide which was developed by the last author (DN) with support from a project steering group, including people with lived cancer experiences and cancer researchers and professionals, and allowed for open discussion with follow-up questions and probing used as appropriate [37].

#### 2.1.2. Theoretical Framework

A theoretical framework for recovery of health and wellbeing in cancer survivorship, based on UK research undertaken with a diverse group of cancer survivors [13], was initially developed in 2011, and it highlights that problematic life events (e.g., cancer diagnosis/treatment) cause an interruption to health and wellbeing, which are also influenced by pre-existing socio-demographic factors. The framework also accounts for the importance of personal (illness perceptions and mental health) and environmental (social support and health service use) factors and how these impact recovery of health and wellbeing, and as such, it was selected to guide the analysis and interpretation of this study to explore how residing in a rural area influences recovery from cancer. The framework posits that cancer-related self-efficacy (confidence to self-manage) is influenced by these problematic events and that engagement with self-management is fundamental to the recovery of health and wellbeing. Whilst the framework accounts for the role of environmental factors (e.g., social support and health service use), there is no explicit reference to rural residency as an environmental factor that could potentially impact recovery. The framework was therefore adapted based on the extant literature on cancer survivorship and rurality. 

### 2.2. Conceptual Framework

First, a conceptual framework was devised by the first (FG) and last (DN) authors using Foster and Fenlon’s theoretical framework [13] described above. 

**Figure 1:**
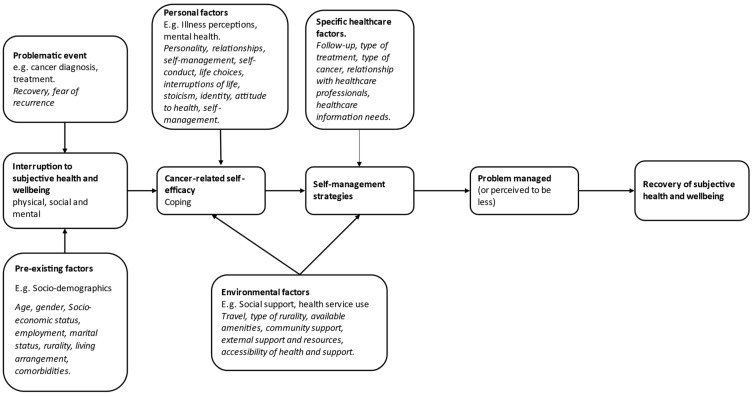
Adapted Framework of Recovery of Health and Wellbeing in Cancer Survivorship, originally by Foster and Fenlon [13].

The framework incorporates the range of factors that can influence each step of the process towards recovery and thus was used to guide and inform data analysis. For example, nodes were created in NVivo (ver. 12) software (QSR International (UK) Limited. London, UK) based on the pre-defined categories from the framework as well by accounting for our own adaptations; for example, some of the key additions included adding ‘recovery’ and ‘fear of recurrence’ to ‘problem incident’, adding ‘travel’ and ‘accessibility of healthcare and support’ to ‘environmental factors’, and adding ‘stoicism’ and ‘identity’ to ‘personal factors.’ Whilst rurality can be considered an environmental factor, it was included as a pre-existing factor within our framework as all of the participants currently resided in a rural area at the time of data collection. The use of the framework to support our analysis meant that we followed a mostly deductive approach with pre-defined categories to be linked with the data. Although the methodological approach was somewhat hybrid in that the first author (FG) had not previously analysed data from our original study, so we wanted to allow for further additions to emerge after familiarisation with the dataset. In that sense, our elaborations and adaptations (shown in italics in Figure 1 below) to the framework were informed by the literature and the dataset itself. 

### 2.3. Ethical Considerations

This paper reports on a secondary analysis of an existing dataset collected as part of a larger study [37], and participants consented for anonymous sharing of their data with other researchers for use in future research. All transcripts were anonymised. This secondary analysis was approved on the 29 October 2021 by the University of Nottingham’s Faculty of Medicine and Health Sciences Research Ethics Committee (REC; Ref: FMHS 371-1021), and a University of Nottingham data management plan was completed prior to the transcripts being shared. The original study was approved by an NHS REC (Ref: 17/WS/0054) on 24 April 2017 as well as by the Health Research Authority (HRA) on 25 April 2017.

### 2.4. Data Analysis

Data from the primary study were available as anonymised verbatim interview transcripts and were sorted to exclude transcripts from interviews conducted with urban participants. The final dataset for secondary analysis consisted of 16 in-depth personal interviews with people who resided in rural areas according to the ONS classifications. Data were analysed using a thematic framework method [24,41] in order to develop themes from both the adapted framework and the data themselves. This approach was used due to its flexibility, allowing coding to be revised to ensure it remained appropriate and allowing inductive and deductive analysis and its accessible and structured method of analysis [39,42].

The last author (DN) had previously conducted all of the interviews, and this secondary analysis was undertaken by the first author (FG). Transcripts were transferred into the Computer-Aided Qualitative Data Analysis Software (CAQDAS) NVivo (QSR International (UK) Limited. London, UK) for reading and analysis. The lead researcher read transcripts multiple times, developed the conceptual framework that was used (Figure 1), and coded the transcripts in NVivo. Inductive coding was carried out initially to derive the codes from the transcripts [41]. Next, the adapted conceptual framework [13] was applied as appropriate to the data. Adaptation used the extant literature prior to coding of data as well as initial familiarity with the transcripts and frequent discussions between the first (FG) and last (DN) author. Codes derived from the framework were reviewed alongside inductive codes in order to avoid any repetition. New codes were input into NVivo, and transcripts were reviewed to apply all codes across the dataset. This is a form of deductive or theoretical coding in which a theoretical framework is the source of the codes and data is fitted into them [43]. A framework matrix was created using NVivo, which collated data from transcripts into their codes within each category. The analysis and data excerpts were combined to present a comprehensive account of the story told by the dataset. Connections between codes and across cases were made using the framework, and were influenced by both the objectives of the research and inductive concepts. Analysis was then written up within the context of the literature in order to interpret the data and its meaning in the context of the wider field of rural cancer research. 

## 3. Results

### 3.1. Demographics

Acknowledging the importance of the pre-existing socio-demographic factors from the adapted Foster and Fenlon framework [13], we report on the specific characteristics of the 16 participants in this secondary analysis in Table 2 below.

### 3.2. Results in Relation to the Recovery of Health and Wellbeing in Cancer Survivorship Framework

Figure 1 presents the modified framework for the recovery of health and wellbeing in cancer survivorship [13]; we structured the results around its core elements. The recovery of health and wellbeing was often facilitated by the support available to participants, which varied depending on the services provided and community in their area, their familial relationships and proximity to family members, follow-up provisions, and ease of access. An explanation of the categories and sub-categories that make up the adapted framework as well as a range of example quotes are reported on below in Table 3.

#### 3.2.1. Problematic Events

The participants tended to have multiple problematic events across their recovery period, and some mentioned that they did not realise how difficult recovery would be until they experienced it. The process of diagnosis was a difficult event for many, with some being shocked by a cancer diagnosis with little warning (*‘that was just a huge shock…cancer had never been discussed’* (Female, 57)) and others finding the build-up or wait time to the confirmation of a diagnosis lengthy and unnecessarily fear-inducing:

*‘Then I had to wait three weeks for the pathology; and when I went back to see her, she said to me that it was a cancer. I just fell apart then because all I had been told was that it couldn’t be a cancer because you have had it so long’*.

(Female, 68)

The treatment took a different form for each participant and interrupted their health and wellbeing in different ways, both physically and psychologically:

*‘[I] tried doing overnight feeds, not very successful. I found that psychologically, I found it very difficult to deal with’* (Male, 62). *‘[due to the chemotherapy] my feet are dodgy and my balance is not very good’*.

(Female, 81)

Recovery post-treatment did not come without its challenges, with many finding that they were not able to operate at their previous normal level: *‘I couldn’t even be bothered to turn the television on…I just hadn’t got the energy…[it was] 12 months really back to the fitness level I was before’* (Male, 69).

A fear of recurrence was identified clearly by the participants as a barrier to the recovery of their wellbeing as it tended to be brought up alongside regular follow-up appointments and thus was never completely gone, leaving patients *‘still conscious, that cancer could come back at any time’* (Female, 68). 

#### 3.2.2. Pre-Existing Factors

The participants often related how their pre-existing factors affected their experiences, describing that the *‘*support for men is pretty dire*’* (Male, 62) and that

*‘[they] are lucky because we are pretty comfortable financially but how on earth someone who’s got what I’ve got [myeloma] that’s just on a pension how the hell they manage I do not know…having to travel there [hospital] everyday’*.

(Male, 69)

For many, family became a vital part of their recovery process: ‘I think I was very lucky because my husband could always take me to the hospital and take me home…I’m lucky I have quite a close family as well. They were always looking after me and cooking’ (Female, 65). Partners and other family members were often relied upon for transportation, encouragement (‘we did it together’(Male, 70)) and emotional support, including when they might have been able to provide qualified advice: ‘My daughter [helped]. She was a big help; being in nursing, she has a lot of insight’ (Male, 81). 

The interruption to health and wellbeing that the problem incident caused varied across the dataset, with some of the attitude that *‘neither of us is getting any younger’* (Male, 75) and others almost feeling that their diagnosis did not affect their life too much: *‘this was something a lot of people my age die with, but not because of it’* (Male, 81).

Comorbidities were a salient concern for some, and some conditions even *‘concerned me more than the cancer really…I was on the verge of type 2 diabetes’* (Male, 81), while others had problems caused by cancer and its treatment: *‘I’ve got an under functioning thyroid, as a result of the radiotherapy…[it] is not going to go away’* (Male, 62). 

Being part of a rural population, every participant mentioned their rurality in one form or another, and this also tied into support in that many described a high level of community support in their area. People felt their rural environment generally improved their wellbeing and described it as *‘idyllic’* (Male, 53), *‘peace and quiet’* (Female, 57), and being able to *‘feel like you can breathe’* (Female, 58). One felt their setting gave them *‘big skies, peace, tranquility…there aren’t any negatives’* (Male, 62).

*‘Wherever you go, and you are driving back, you think, “I live here. It’s wonderful.” When you go with the walking group, they all say they are lucky living here. It is completely different here. It is very unthreatening…It gives you an enormous feeling of wellbeing; being here. Just being in this town is a very satisfying place; everybody says hello and smiles. Everybody in this close here knows each other and is friendly. We all have a meal together at Christmas in town; and we are all different ages’*.

(Female, 68)

#### 3.2.3. Personal Factors

A culture of stoicism was also shown within the data, with many describing that they *‘just get on with it’* (Female, 55), utilising little formal support and using their own attitudes and strategies to recover their wellbeing. This links into attitudes towards health, self-management, and identity in that these attitudes were highly used by participants throughout their survivorship experience. Descriptions of being *‘pragmatic when it comes to illness’* (Female, 58) and *‘drawing on some techniques that I have picked up…to make sure that I can hack this’* (Female, 57) as well as having a *‘positive outlook’* (Female, 39) were common in various forms across the dataset. These can-do attitudes and pragmatic approaches meant that some of the participants had a high degree of cancer-related self-efficacy and were mostly confident when it came to engaging with self-management strategies and behaviours. Linking into the interruption of subjective health and wellbeing, the participants often found that their experiences with cancer gave them a sense of altered identity or that they took on another identity of being the *‘sick one’* whilst undergoing treatment and finding their new normal in life. 

Although the majority of the participants were retired, some found that work stayed a strong part of their identity, even using it as a self-management strategy for getting back to what they felt was their normal: *‘I went back to work, after treatment, in hindsight, probably a bit too quickly, I was very keen to get back to work and get back to normality’* (Female, 39).

The participants frequently spoke about how they had a period of being not up to their previously normal activities of daily living (ADL) during their recovery, taking on a different role in their life before finding normality again, and felt that *‘you are spending 18 months after the operation like you feel you can’t do anything’* (Female, 55), but once that period had passed, *‘I thought, once it was done it was done and that was it. As if like, breaking a leg, I thought “The leg’s mended, be careful for a while and now we are off again.” I didn’t think about it’* (Male, 70).

Some described an emotional journey they went on due to the life-changing impact of cancer on their and their families’ lives: ‘I also had to sort of then deal with the fact that I can’t have children, the emotional impact of that. I had the menopause, that had massive implications, and also within our marriage, things had changed, both of us trying to get our heads around that and where we wanted to go in the future, did we want to adopt kids, all that kind of stuff. So there were so many different things, so I did have a really tough time mentally’ (Female, 39). Others were very stoic and found they could just move on with their lives as if nothing had happened to them: ‘Once the procedure was over, I thought “That’s it, its either fixed or it isn’t, now let’s get on.” I didn’t really give it a second thought. I’ve never actually sat and thought, “Oh you know what, I’ve had prostate cancer”’ (Male, 70).

Stoicism ties into the varied attitudes towards health the participants had and some were fairly nonchalant and accepted their cancer experience as a part of life (*‘It has not really affected my life…I thought, “Yeah, it’s something that people get”’* (Male, 70)), whereas others found it harder to cope with: *‘I hate being ill, hate being ill’* (Female, 68).

#### 3.2.4. Environmental Factors

Many found that their rural setting had a high level of community support, expressing how ‘if anything goes wrong, you know that you can just run down the lane and there will be somebody there that can help you out’ (Female, 58), and that ‘it is quite a supportive environment’ (Male, 53). Having social support in a close proximity influenced their cancer-related self-efficacy and some of the participants’ engagement with self-management practices and strategies. 

Access to healthcare was frequently mentioned as being rural, the participants often had to travel long distances to hospitals and other services, finding that *‘it was probably four or five hours out of their day every day. And so it does make you think, “There should be closer facilities, why aren’t these facilities available at [local town] hospital?”’* (Female, 58), and some found that the travel had a direct detrimental effect on them:

’*I think the other psychological effect at the time was the journey to and from [hospital] 80, 90 miles. And the appointments were never consistent, you know I could have a late afternoon one and be there for eight o’clock the next morning. That was not great’*.

(Male, 62)

Several of the participants were helped in a way similar to familial help by their communities: ‘In the village itself, I made friends that year really within walking distance which is nice, so there was always someone around. Very rarely did I have a day go by where I didn’t have a visitor just popping in to check I was all right’ (Female, 39). Others, however, found support available to them in different forms, such as community groups (‘The golf club has been part of my salvation as well. It is every Tuesday the ladies play. Even if I didn’t want to play, there is a great crowd of people there. It is a bit of a life line really. They are all interested in my situation’ (Female, 81)), online forums (‘The website was Beating Bowel Cancer. It was factual; explained things. I found it helpful after my reversal’ (Female, 62)), and their local GP surgery (‘[I could] phone the doctor and he’d say, “I’ll just pop out the surgery” that is just unheard of in lots of ways so the doctor would go and just come out and just knock on the door and come and have a look’ (Male, 53)).

As the nature of being rural is not living in major towns and cities, where hospitals tend to be situated, travel was one of the main points in discussions around rurality. Travelling long distances to services was considered by some participants to be a trade-off for living in a rural area, being described as *‘a choice you make, you have to weigh this up when you choose to live somewhere like this and think clearly, “Is it accessible for hospitals and things?”’* (Female, 58), but other support was nearby, such as GP surgeries, so residents *‘don’t feel disconnected from the opportunities to ask questions so, the doctor down the road is really good’* (Male, 53).

Healthcare resources, or a lack thereof, were mentioned alongside experiences of healthcare professionals and settings, and they even detrimentally affected the wellbeing of some participants: *‘I had to wait over the weekend, and into the following Thursday until a radiographer could come and do it…I was in a lot of pain’* (Male, 62).

#### 3.2.5. Specific Healthcare Factors

Specific healthcare factors were also important in the recovery period as they influenced how quickly ADLs and subjective health and wellbeing were recovered and how quickly self-management was achieved. For example, participants said, *‘I just feel that cancer…the spectrum is like, from nothing which is mine, to horrific’* (Female, 58), and gave descriptions of their ability to work: *‘I could not have carried on working when I was having chemo. I was just so ill’* (Female, 65) compared to *‘for the immediate period after the procedure, the week I didn’t work. But then within a week I was back at my desk’* (Male, 70).

For many, the cancer experience was largely shaped by their experiences with healthcare professionals and settings and the treatment they received, which varied according to access to hospitals, type of treatment, personal attitude to healthcare, and healthcare information needs.

The type of treatment depended on the type of cancer each participant had and ranged from very minor (*‘I had a chunk chopped out’* (Male, 53)) to major treatment pathways (*‘*a mastectomy and three weeks of radiotherapy…[then] I had to go onto chemo’* (Female, 81)). This affected the participants’* coping strategies, self-management, and time taken to find themselves at a point of recovering their subjective health and wellbeing.

Each person had a unique experience of hospitals, healthcare professionals, and treatment regimens, and as such, these shaped how each person felt about their situation and how well they coped. Most had very positive experiences (*‘I would say the Macmillan nurses and the prostate nurse at the [hospital]; we can’t fault them and the help they have given us’* (Male, 75)), but a small minority had trouble and felt they were negatively affected going forward because of it: *‘The hospital was shocking, [hospital X] the breast unit and that’s what set me up now for the problems that I think I have had later on…it was a really bad experience’* (Female, 55).

For some, few follow-ups were provided to them (or less than anticipated), and cancer support groups were not available in their area; one participant found that when it came to support groups, *‘there is nothing round here…they are miles away, absolutely miles away’* (Male, 62), and another described how they felt after completing treatment:

*‘It is funny after you have had all the treatment, and then all of a sudden it’s “See you in a years time”…but you do feel like all of a sudden you have had so much care, and then abandoned’*.

(Female, 65)

## 4. Discussion

### 4.1. Key Findings

This study provides in-depth insight into the experiences of rural cancer survivors. The findings show that the whole cancer experience is influenced by a range of personal, demographic, and environmental factors and that for the participants, these influences were positive as often as they were negative. Rurality had a variety of impacts on their recovery experiences, including providing high levels of community support, a natural sense of wellbeing, close relationships with GPs, a culture of stoicism, a considerable distance to travel to healthcare services, a lack of availability of diverse structural support, and a feeling of disconnection from specialised healthcare providers. We presented empirical data to demonstrate how the key components of the applied theoretical framework [13]; problematic events; pre-existing, personal, and environmental factors; as well specific healthcare factors can interrupt subjective health and wellbeing and in turn influence cancer-related self-efficacy and self-management strategies. This is the first study of its type carried out in the East Midlands of England, and as such, it reveals contextual details of the cancer experience in this region. Much of the current qualitative research available on the experience of cancer survivorship is based in the US or Australia, and as such, this study adds to the growing exploration of cancer recovery and survivorship across the UK. We believe this is the first time the framework, initially developed by Foster and Fenlon in 2011 [13], has been used in rural cancer survivorship research and applied solely to a rich qualitative dataset. The utility of the adapted framework in this study demonstrates its value as a tool for use in the field of qualitative cancer survivorship research. As the adapted framework is not entirely specific to rurality, it could also be applied to wider studies in this field as support and other factors have been shown to be more important in the survivorship experience than rurality itself.

### 4.2. Findings in Context of the Academic Literature

It has been identified across the literature that more specific research on cancer survivors and their needs is required, especially in rural populations [5,31,44]. The results are consistent with many reports of the positive aspects of rurality, such as high levels of community engagement and support [28] and the high self-efficacy found in the surveys conducted in our wider study [35]. The other factors contributing to the survivorship experience, which was previously found to have an effect on cancer survivors, have been mirrored by these results, and these include social support, practical challenges, access issues, and persistent symptoms [9,18,20,33]. 

Pre-existing factors, such as gender and SES, have been shown to affect cancer outcomes and experiences [15,45], and our analysis highlights examples of this. Both personal and environmental factors feed into how people cope and into their cancer-related self-efficacy, and it has been found that rural populations experience higher levels of community support and have generally higher self-efficacies [18,35]. This is reflected by the positive experiences of communities in rural areas in this study, finding that most participants relied on community support to support their coping and self-management skills. A culture of stoicism is often identified amongst rural populations [19,34,46], and was shown within the current data, with many describing that they just *‘get on with it’*, relying little on formal support and using their own mental resources and strategies to recover their wellbeing. Difficulty accessing healthcare and support is often observed amongst rural populations [18,19,26,27,28,29,30,47], and the current findings seem to confirm this, with many not accessing services, such as support groups, due to distance, a lack of local specialised treatment services, and the direct effects of travel on their wellbeing.

### 4.3. Strengths

The adaptations made to Foster and Fenlon’s framework [13] and coding presented in this study were discussed over several meetings between the first (FG) and last author (DN), the latter of which is an experienced qualitative researcher with a doctorate in cancer survivorship, and as such, the adaptations were corroborated and validated. The adaptations to the framework were informed by these discussions as well as the extant literature on cancer survivorship and rurality. Across the analysis period, the lead researcher ensured she reflected upon the dataset and emerging themes, noting ideas and thoughts in a notebook for reference as is encouraged throughout the process [48,49]. Initial codes, subsequent findings, and accompanying narratives were then validated over several meetings with the last author (DN), who conducted the original interviews.

### 4.4. Limitations

A key limitation of this study is that the thematic analysis was carried out by only one researcher due to time constraints. However, the use of inter-rater reliability is not agreed upon by all proponents of thematic analysis, such as Braun and Clarke, who suggest individual reflexivity should suffice, making multiple coders unnecessary [50,51]. It could be argued that the sample size within this study is small and thus lacking in breadth; however, the analysed interviews contain rich and detailed narratives from individual participants, providing a considerable volume of data for analysis. As the interviews were conducted only within the East Midlands of England, the results may have limited applications across the rest of the UK and internationally due to the widely ranging population densities and different definitions and conceptualisations of rurality between countries [52,53]. This may, however, be a useful starting point for other studies as the framework could be applied to similar qualitative studies in the field of cancer recovery. The demographic of this study was neither ethnically nor socially diverse, being completely white and mostly retired, meaning it is likely that there are perspectives not reflected within the data. Future studies could use purposive sampling in order to capture a more diverse set of experiences.

### 4.5. Implications and Suggestions for Future Research

The findings suggest that whilst rurality is a contributing factor, there might be other things at play beyond rural residency that can support the survivorship experience, such as community support and the variety of pre-determined and personal factors contributing to self-management and coping skills. Furthermore, future research should evaluate how age can play a role regarding attitude towards health, interpretations of life, self-conduct, and self-management. The adapted framework used in this research could be applied to other datasets on cancer survivors as the majority of the factors are not necessarily unique to the rural environment. The application of the framework to a similar qualitative dataset with solely urban participants would make for an interesting comparison to reinforce or challenge some of our findings. These results have the potential to support the development of targeted interventions with rural cancer survivors although before this can happen further confirmatory studies are warranted. Future research is necessary to understand how this set of factors affects the wider cancer survivor population in both rural and urban settings across the rest of the UK and indeed internationally. The interviews and analysis could be replicated across different settings to gain a broader understanding of peoples lived experiences when recovering from cancer. The emerging themes could be employed in decision-making regarding care for rural populations, providing insight into their cancer experiences. 

### 4.6. Conclusions

This study is novel in that it applies a well-established framework [13] to a rich qualitative dataset on post-treatment cancer survivors. It shows that residing in a rural area can have a positive and negative impact on one’s recovery following treatment through its influences on other contributing factors across the process. The identified barriers to post-treatment care are lengthy travel times and distances, a lack of availability of local specialised support services, as well as a feeling of abandonment by the healthcare system. The opportunities available to cancer survivors in rural areas are strong community support and ties, a feeling of heightened wellbeing in one’s setting, high levels of independence and stoicism, and good self-management. It is suggested that rurality itself is not necessarily the deciding factor in cancer recovery; rather, the range of other factors intersecting with it all play a part in survivors’ experiences. Future practitioners and policy makers should consider working with local communities to tailor support to the specific characteristics of the rural environment.

## Figures and Tables

**Table 1:** Participant eligibility criteria.

*Inclusion*	*Exclusion*
*≥18 years old*	<18 years old
*Diagnosis of cancer and registration in the database of cancer patients at largest acute NHS Trusts at the study sites*	Metastatic spread and/or evidential cancer recurrence
*Have undergone primary cancer treatment up to 5 years ago*	Cancer treatment initiated within the last year
*Willing and able to give informed consent*	Currently under palliative care
*Adequate level of spoken and written English*	Spoken and written English not at adequate level

**Table 2:** Socio-demographic profile of participants.

	All Respondents (*N = 16*)	*n* (%)
*Age (at start of study)*	35–44	1 (6.3)
	45–54	1 (6.3)
	55–64	7 (43.7)
	65–74	5 (31.2)
	Over 75	2 (12.5)
*Gender*	Female	10 (62.5)
	Male	6 (37.5)
*Living arrangement*	Partner/spouse/family/friends	13 (81.2)
	Alone	2 (12.5)
	Missing data	1 (6.3)
*Marital status*	Married/civil partnership	12 (75.0)
	Living with partner	1 (6.3)
	Widowed	2 (12.4)
	Missing data	1 (6.3)
*Employment status*	Employed	3 (18.7)
	Retired	10 (62.5)
	Other	2 (12.5)
	Missing data	1 (6.3)
*Annual household income*	£0–14,999	2 (12.5)
	£15–24,999	3 (18.8)
	£25–49,999	5 (31.1)
	£50–74,999	1 (6.3)
	Over £75,000	2 (12.5)
	Missing data	3 (18.8)
*Residence—degree of rurality*	Rural town and fringe	6 (37.5)
	Rural village	6 (37.5)
	Rural village in sparse setting	1 (6.3)
	Rural hamlet and isolated dwelling	3 (18.7)
*Primary cancer type*	Breast	4 (25.0)
	Urological	3 (18.7)
	Skin	2 (12.5)
	Head and neck	3 (18.7)
	Gynaecological	2 (12.5)
	Lower gastrointestinal	1 (6.3)
	Haematological	1 (6.3)

**Table 3:** Contributing factors in the recovery of subjective health and wellbeing during cancer recovery.

Category	Subcategory	Explanation	Example
Problematic events	Cancer diagnosis	The process of a diagnosis of cancer	*‘I had gone in and never thought for one minute that I had got cancer…I got taken into the room with a consultant and the Macmillan nurse. And she just said straight away, “Well, I’m sorry, you have got cancer.” Well…you think the worst don’t you…that was just a huge shock…cancer had never been discussed.’* (Female, 57)
	Cancer treatment	Any part of the treatment process	*‘So I had surgery and twenty three lymph nodes removed; of which eighteen were fine. The others were showing signs of having cancer. It was awful.’* (Female, 65)
	Cancer recovery	Any issues or obstacles following treatment	*‘I hadn’t appreciated quite how long the side effects, in terms of tiredness and energy levels were going to continue for.’* (Female, 39)
	Fear of recurrence	The worry that cancer will return	*‘You get really obsessed about it coming back. Or every ache and pain—“Is that cancer!”’* (Female, 39)
Pre-existing factors	Age		*‘One of those things that most people my age when they get to their 60s and 70s they have got these cells in their bloodstream and most people live with it harmlessly.’* (Male, 69)
	Gender		*‘Support for men, is pretty dire, pretty dire.’* (Male, 62)
	Marital status	Partnership or lack thereof, divorce, widow/er	*‘I am married and have been for 40 years.’* (Female, 62)
	Living arrangement	Who the participant lives with	*‘My house is too big for me; but I’ll stick here until they carry me out.’* (Female, 81)
	Socioeconomic status	Household income, employment status, housing, resources available to the participant	*‘I am just fortunate in my lifestyle um [pause] which means I don’t have to have those added pressures you know I don’t have to earn money because I have done that in my earlier life which is now set me up for my later life.’* (Female, 55)
	Comorbidities	Any other conditions the participant has	*‘I’ve got labrynthitis, occasionally my ear becomes infected and I get sick and fall over.’* (Female, 68)
	Familial support	Available help from family members	*‘My mum in law used to come a lot with me so I could remember everything that we discussed.’* (Female, 39)
	Employment	Whether or not the participant is in work and how they perceive it	*‘Being freelance there is no “If I don’t work”; you know you don’t work you don’t earn sort of thing.’* (Male, 53)
	Rurality	Setting they live in	*‘Very flat, very rural, largely agricultural. Little in the way of large settlement. This village is very quiet indeed.’* (Male, 62)
Personal factors	Interpretations of life/self-conduct	How the participant views life and how they carry themselves	*‘I think when you get older, you get things in perspective a little easier, than when you were younger.’* (Female, 58)
	Identity	How the participant views themselves, the activities they enjoy, and their personality	*‘I’ve always been an organiser, that has kept me going and I do a lot of organising.’* (Female, 68)
	Stoicism	Continuing through adversity in life with no complaint or outwardly viewable suffering	*‘I’ve survived on my own and coped quite well.’* (Female, 81)
	Attitude to health	How the participant views their health and looks after it	*‘I just think if your number is up, your number is up.’* (Female, 58)
	Self-management	How they look after their own health, behaviour, and lifestyle	*‘Self-management is about I guess, taking ownership of your own body and learning that you can impact it yourself, by taking care of yourself in different ways.’* (Female, 39)
Environmental factors	Travel	Public transportation, travel by car, and distance from amenities	*‘There are no buses down here, you either have to get a car, or walk.’* (Female, 57)
	Type of rurality	How dispersed the population is	*The UK Office for National Statistics (ONS) RUC2011 Rural Urban Classification (e.g., Rural Village in a Sparse Setting)*
	Community support	Available help from others in the local area and local recreational activities	*‘If anything goes wrong, you know that you can just run down the lane and there will be somebody there that can help you out.’* (Female, 58)
	Access to support	How available structural support, such as healthcare professionals and support groups, is to the participants	*‘There is a doctor surgery at the village we are in.’* (Male, 53)
	Healthcare resources	Facilities, personnel, and funds for healthcare provision (or lack thereof)	*‘There was a severe shortage of beds.’* (Male, 70)
Specific healthcare factors	Follow-up	Any contact with healthcare staff after treatment	*‘I had six week reviews. They were later moved up to three months.’* (Male, 62)
	Type of treatment	What sort of intervention the participant had for their cancer	*‘Then I went for chemo, radiotherapy for 28 days.’* (Female, 62)
	Type of cancer	The specific diagnosis	*‘Stage 2 bowel cancer.’* (Female, 62)
	Relationship with healthcare professionals	The trust, belief, and opinions that the participants have in clinicians	*‘There has been the occasional person I have not been happy with, but on the whole for my cancer treatment, they have been very good.’* (Female, 68)
	Healthcare information needs	Any need felt by the participants for more knowledge of their condition, treatment, or health status	*‘There isn’t really that much information about how to manage yourself going forward.’* (Female, 39)

## Data Availability

The anonymised data are available upon reasonable request by contacting the corresponding author.
